# *Cherub* versus *brat*

**DOI:** 10.7554/eLife.36030

**Published:** 2018-03-27

**Authors:** Jennifer A Malin, Claude Desplan

**Affiliations:** Department of BiologyNew York UniversityNew YorkUnited States

**Keywords:** tumorigenesis, lncRNA, neuroblasts, neural stem cells, cherub, brat, *D. melanogaster*

## Abstract

A long non-coding RNA molecule called *cherub* is a driver of tumor development.

**Related research article** Landskron LK, Steinmann V, Bonnay F, Burkard TR, Steinmann J, Reichardt I, Harzer H, Laurenson AS, Reichert H, Knoblich J. 2018. The asymmetrically segregating lncRNA *cherub* is required for transforming stem cells into malignant cells. *eLife* **7**:e31347. doi: 10.7554/eLife.31347

How healthy cells become tumors is a question that has interested scientists for decades. Many tumors exhibit genomic instability – that is, increased numbers of DNA mutations and chromosomal rearrangements. However, some tumors – including most childhood cancers – have few mutations and are thought to be driven by mechanisms other than genomic instability (Lee et al., 2012).

Important cancer research in flies has been carried out using neural stem cells called neuroblasts. Normally, a type of neuroblast called a Type II neuroblast divides asymmetrically to form a new neuroblast and a slightly more specialized cell type called an intermediate neural progenitor cell (INP); the new neuroblast is able to repeat this process several times. In parallel, the INP continues to divide to produce a new INP and a neuronal precursor cell in each round (Bello et al., 2008; Boone and Doe, 2008; Bowman et al., 2008, Figure 1A). The neuronal precursor then divides to generate mature neurons. When a tumor suppressor gene called *brat* is blocked, INPs transform back into tumor neuroblasts that divide indefinitely (Lee et al., 2006). So far, the mechanisms involved have remained unclear.

**Figure 1.**
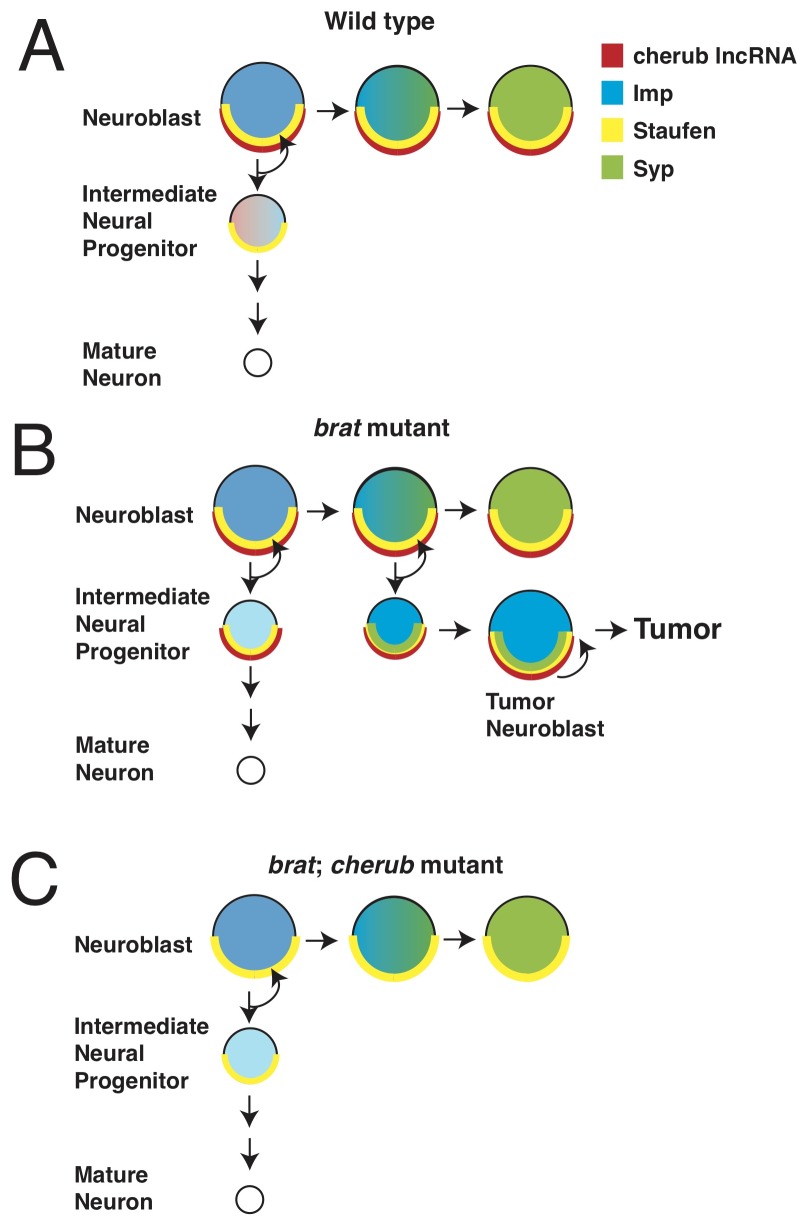
Misregulation of *cherub* RNA causes tumor growth. Neurons develop from neural stem cells called neuroblasts and specialized cells called intermediate neural progenitor (INP) cells. Landskron et al. found that a long non-coding RNA (lncRNA) named *cherub* has a role in the development of brain tumors. (**A**) In healthy flies, the protein Staufen (yellow half circle) anchors *cherub* (red half circle) to the basal part of the neuroblast cell membrane. After the neuroblast divides, *cherub* is inherited in the INP cell and spreads within it. As neuroblasts mature, they start to produce the protein Syncrip (Syp: green), which reduces the levels of a protein called Imp (blue). (**B**) In flies that lack the tumor suppressor gene *brat*, *cherub* remains at the cell membrane of the INP cells. When Syp is expressed as the neuroblast ages, it also remains anchored to cell membrane of the neuroblast by *cherub* and Staufen. This prevents Syp from repressing Imp, and some of the INP cells develop into tumor neuroblasts. (**C**) In flies that lack *brat* and *cherub*, *cherub* can no longer tether Syp; this allows Syp to inhibit Imp, and the neuroblasts are able to mature.

Now, in eLife, Jürgen Knoblich and colleagues at the IMBA in Vienna and the University of Basel – including Lisa Landskron as first author – report that genomic instability is not a driver of neuroblast tumors (Landskron et al., 2018). Landskron et al. compared healthy flies and flies in which the *brat* gene had been mutated and showed that both groups of flies had the same number of chromosomes. Moreover, they found no additional DNA mutations responsible for tumor development.

However, further analyzes revealed that the tumor cells had increased levels of a previously uncharacterized long non-coding RNA: this is a molecule that is transcribed from DNA but is not usually translated into protein. Landskron et al. found that this specific long non-coding RNA, which they named *cherub*, was not required for normal development, as mutant flies lacking *cherub* were healthy and fertile. However, flies with a defect in both the *brat* gene and the *cherub* gene had smaller tumors, which suggests that *cherub* is required for tumor progression.

While other long non-coding RNAs have been implicated in tumor growth, *cherub* is the first to be discovered that is not uniformly distributed throughout the cell. Rather, *cherub* is located at the basal end of the neuroblast cell membrane. When healthy neuroblasts divide, *cherub* is distributed into the INP, which forms on the basal side of the neuroblast, and then diffuses throughout the cell. Thus, with each round of division, *cherub* levels slowly decrease in the INP (Figure 1A). In *brat* mutants, however, *cherub* remains at the cell membrane in both the neuroblast and in the INP. This faulty localization prevents the dilution of *cherub* and causes the INP to transform back into a tumor neuroblast (Figure 1B).

Why does the incorrect localization of *cherub* cause tumor growth? In fly neuroblasts, the proteins Imp and Syncrip regulate neuroblast aging (Ren et al., 2017). Normally, these two proteins inhibit each other: Syncrip levels increase as the neuroblasts age, while Imp levels decrease. Both the expression of Syncrip and the absence of Imp are required for neuroblasts to stop dividing (Yang et al., 2017, Figure 1A).

Landskron et al. showed that in flies lacking *brat,* Syncrip and *cherub* are inappropriately localized at the tumor neuroblast cell membrane. Moreover, Syncrip and Imp are both expressed. This is presumably because newly-produced Syncrip remains tethered to *cherub* at the cell membrane, where it is unable to reduce Imp levels (Figure 1B). Thus, the tumors continually produce Imp and do not age. When *cherub* is not present, Syncrip remains distributed throughout the cell. This reduces the levels of Imp which, in turn, slows the growth of any tumor and improves the life expectancy of *brat* mutant flies (Figure 1C). How does *cherub* localize to the cell membrane? The experiments revealed that *cherub* binds to another protein called Staufen, which attaches to the asymmetric cell division machinery, and thus tethers both *cherub* and Syncrip to the cell membrane.

This work reinforces the notion that changes to the proteins that regulate asymmetric cell division and cell fate can drive tumor formation. Previous research has shown that Imp levels are also dependent on another protein called Chinmo, and that overexpression of Chinmo causes tumor growth by maintaining inappropriately high Imp levels during the window when Imp is normally expressed (Narbonne-Reveau et al., 2016). Thus, preventing stem cells from maturing and aging appears to fuel tumor development.

This study also highlights the importance of long non-coding RNAs in stem cell development and in tumor formation. While it has been shown that other long non-coding RNAs are involved in stem cell development, this may be one of the first examples where changing the localization of such molecules prevents stem cells from aging, thus driving tumor growth (Guttman et al., 2011). It will be interesting to see whether other long non-coding RNAs use the same mechanisms to drive tumorigenesis.
